# Deaf client with bipolar illness: a case report

**DOI:** 10.1186/1745-0179-3-19

**Published:** 2007-09-28

**Authors:** Maneesh Gupta, Jenny Caddy

**Affiliations:** 1 Camborne Community Mental Health Team, Josiah Thomas Memorial Hall, Cornwall Partnership NHS Trust, Cornwall, TR14 8LQ, UK

## Abstract

**Background:**

This case report highlights the diagnostic and assessment difficulties faced by mental health professionals when dealing with a Deaf client.

**Case presentation:**

We used mobile phone text facility to monitor and liaise with the client while in the community. We focused on the affect and signing amplitude/intensity of the client to make a diagnosis of bipolar disorder, prescribed valproate semisodium, and noticed an improvement in two months.

**Conclusion:**

This is an example of some areas of good practice when assessing a Deaf client with mental health problems.

## Background

Deaf clients with mental health problems pose substantial challenges to mental health professionals. The diagnostic and assessment process is skewed towards using verbal communication and expression, which might be difficult in Deaf clients. Social problems also arise due to the communication difficulties with neighbours and local organisations.

We present a case report of a successful outcome in a client who was provided services by our Community Mental Health Team (CMHT).

## Case presentation

Mrs AB is Deaf. She communicates using British Sign Language (BSL). She was referred to us by her General Practitioner (GP) because of his concerns that she was hearing voices. She had been apparently well over the last 5 months on an antidepressant. The GP had prescribed a benzodiazepine and provided us with information on the availability of a BSL interpreter.

### Assessment

An urgent assessment revealed that the client "may be having psychosis" and continued support was advised by the CMHT.

In this assessment, the discrepancy between the subjective low mood and objective cheerful affect was mentioned. The client was pleasant, cheerful, rate pressured, thoughts mostly of anger towards social services. The voices were described as outside her head, and of GOD wanting to kill her.

The Community Psychiatric Nurse (CPN) (JC) who was allocated the client assessed her with a BSL interpreter. She also used text and type talk to interact with and monitor the mental health of the client. The experience of using text to communicate with the client was better than using type talk. Type talk took longer and it felt devoid of a personal rapport with the client. Text was faster, did not bind you to the phone and was more personal. In the two months that we were involved, text was used 8–9 times (each time a series of text messages were used to seek or provide information, reassure, confirm appointments etc), while type talk was used only twice. The client preferred text and mentioned type talk is "boring" and it "takes a lot of time"

### Past history

Since the client had been known to mental health services in her previous area of stay, her notes were received and reviewed. The client had been diagnosed as depressed and had a brief inpatient stay in an inpatient unit for Deaf clients with mental health problems. Mood instability, anger and different voices were noted. On one instance, Mrs AB had spoken about one or two voice(s) which may have been inside or outside her head, which were playing music and were interfering. On another occasion, she had mentioned hearing God's voices as well as clapping, people partying, and enjoying themselves. There was also documentation of voluble signing and of ongoing problems with neighbours.

### Outpatient Assessment

When she was assessed by the Consultant Psychiatrist (MG), she had rapid signing with increased amplitude, along with intense facial expressions, lip and tongue movements and attempts at speaking. She was difficult to interrupt, and repeated an incident in the last 48 hours with great emotional involvement. The predominant theme was of anger towards her neighbours and of being blamed for how her children were (they had behaviour disturbances). There was evidence of flight of ideas. A provisional diagnosis of bipolar affective disorder, hypomania (F31.0) was made and valproate semisodium advised. After a few weeks, during which the CPN interacted and supported her, she managed to be consistent with the valproate semisodium. At 750 mg she accepted that the voices had gone and her son expressed "mum has improved"! We noticed that her signing was calmer than and not as intense as before.

### Outcome

She continued on 1000 mg valproate semisodium for a few weeks and felt "so much better" that she decided to stop the medication. 5 weeks later, she began feeling angry at the situation around her and expressed frustration at her family and friends. At assessment she was found to be presenting with similar symptoms as initially, and was advised to restart valproate semisodium. She continues to improve. CPN support is mainly by text, augmented with planned visits with the interpreter.

## Discussion

Not all Deaf patients with mental health problems are provided support by the tertiary deaf mental health services. The local CMHT is the first to assess such clients for mental health problems. Without adequate training or experience of dealing with Deaf clients, it is for individual teams to innovate and provide the best possible diagnostic and interventional care for such clients.

This case report highlights a few areas of good practice.

1. If the referring GP can provide information for the CMHT about the availability of a BSL interpreter, it is easier and faster to get the first assessment done. It is important to choose a skilled interpreter who is comfortable in interpreting when a Deaf person may be having mental health problems.

2. Using modern technology e.g. text facility on the mobile phones, a CPN can monitor and support Deaf clients easily and faster than with type talk. This is used in consonance with face to face support with BSL interpreter.

3. When a diagnostic assessment is carried out, it is essential to document the speed and intensity (amplitude) of signing by the client. It is also important to explore the discrepancy in mood and affect, if present; and to document the nature of voices. If we merely focused on what the client is telling us, we might miss important markers to a diagnosis. As with all psychiatric disorders, the entire presentation and the change in the client's presentation should be of diagnostic importance.

4. In the assessment process, it is essential to document in detail about the voices which the client may report to be hearing. Discrepancies in the presentation of "voices" may be found in Deaf clients.

5. Anger and mood instability in a Deaf client with apparent mental health problems might respond to valproate semisodium.

Despite a growing recognition of mental health problems in Deaf clients, there is very little published evidence on approach to diagnosis and interventions. A brief report in 1999 [1] pointed out the high rate of misdiagnosis in Deaf clients despite an inpatient admission. Of 28 inpatients over a 15 year period in a psychiatric inpatient unit, 11 had bipolar. However, these clients were diagnosed correctly only in 7 (29.2%) of 24 inpatient episodes.

Thacker wrote about errors in British Sign Language communication when Deaf people were suffering from schizophrenia [2]. Haskins [3] mentions the presence of a "hand shape neologism" in a patient suffering from schizophrenia. One case report [4] mentions "flight of fingers" in a congenitally Deaf person with mania, using American Sign Language. However, a diagnostic assessment format and criteria for diagnosis of mental illness in Sign Language users has not been attempted.

Mental health professionals in specialised Deaf mental health units need to be proactive in generating such evidence for diagnostic assessments and therapeutic interventions.

## Conclusion

This case report provides examples of good practice when assessing a Deaf client with mental health problems.

## Competing interests

**Dr Maneesh Gupta **has accepted hospitality and remuneration for speaking, from various pharmaceutical companies. Dr Gupta holds some shares in Indian pharmaceutical companies.

**Mrs Jenny Caddy **has accepted hospitality from a pharmaceutical company.
